# Dialoguing with algorithms: experiences of generative AI use in navigating complex health decisions among older adults with multimorbidity: a qualitative study

**DOI:** 10.1186/s12913-026-15158-x

**Published:** 2026-07-23

**Authors:** Qin Lin, Mengxue Fu, Pei Chen, Jiaorong Zhao, Yanhua Liu, Yanping Niu, Minmin Jiang, Jijun Wu

**Affiliations:** 1 Shulan International Medical College, Zhejiang Shuren University, Hangzhou, Zhejiang 310015 China; 2 Department of Rehabilitation, People’s Hospital of Jianyang, Jianyang, 641400 China; 3 Division of Health, University of Waikato, Hamilton, 3240 New Zealand; 4 Nursing Department, People’s Hospital of Deyang, Deyang, 618099 China

**Keywords:** Aged, Multimorbidity, Generative AI, Complex health decision-making, Psychological experience, Technology acceptance model, Qualitative research

## Abstract

**Background:**

Generative AI has reshaped healthcare, yet multimorbid older adults face digital divide barriers limiting its safe application. Existing qualitative evidence on their AI-assisted complex health decision-making remains scarce, lacking integrated theoretical interpretation.

**Objectives:**

Guided by an integrated framework combining the Technology Acceptance Model, Senior Technology Acceptance Model and Burden of Treatment Theory, this study explored older adults’ perceived usefulness and ease of generative AI for health decisions, mobilizable personal-social resources, and how technology perceptions shape dynamic usage behaviors and decision quality.

**Methods:**

From September to December 2025, 16 multimorbid seniors were recruited via purposive maximum variation sampling from an urban community health center in eastern China. Face-to-face semi-structured interviews were conducted, with reflexive thematic analysis following COREQ guidelines.

**Results:**

Based on interviews with 16 participants, four themes were identified: (1) Perceived usefulness, which was mainly reflected in four dimensions: initial situational triggers prompting AI consultation, convenient access to information, integrated support for multimorbidity management, and enhanced decision-making confidence. (2) Perceived ease of use: Participants’ perceptions of the ease of use of generative AI showed significant individual differences and context dependence. (3) Resource mobilization: Successful use of generative AI rarely depended on independent individual effort but required the mobilization of diverse personal resources and social support. (4) Behavioral intention and actual use: The generative AI usage behavior of older adults with multimorbidity exhibited dynamic evolutionary characteristics; its trajectory was not linear acceptance and continuous use, but a complex process involving attempts, interruptions, resumption, or permanent abandonment.

**Conclusions:**

This study reveals that AI use among older adults with multimorbidity is not merely a technology adoption behavior, but an adaptive strategic choice made under the pressure of managing multiple chronic conditions. Only by fully considering the special needs of patients with multimorbidity in technology design and service provision can the safe and effective application of generative AI in elderly chronic disease management be truly realized.

**Trial registration number:**

Not applicable. This study did not involve clinical trials.

**Supplementary Information:**

The online version contains supplementary material available at https://doi.org/10.1186/s12913-026-15158-x.

## Introduction

Multimorbidity in older adults refers to the coexistence of two or more chronic diseases in an individual, which has become a major public health challenge facing the global aging society [1]. With the accelerating process of population aging in China, the prevalence of multimorbidity among people aged 60 years and older has exceeded 50%, showing a significant upward trend with advancing age [2]. Such patients not only need to cope with complex symptom management and polypharmacy issues, but also frequently face dilemmas in complex health decisions, including cross-specialty coordination and trade-offs among treatment regimens [3]. The traditional fragmented healthcare model struggles to meet their demand for integrated care, leaving patients often in a passive situation characterized by difficulties in accessing information, low participation in decision-making, and insufficient self-management ability, which further affects treatment outcomes and quality of life.

The rapid development of generative artificial intelligence has brought revolutionary changes to the healthcare field. Large language models represented by ChatGPT, DeepSeek, Kimi and others demonstrate strong natural language understanding and generation capabilities, which can provide users with real-time, personalized, and multimodal health information support [4]. In terms of health decision support, generative AI can integrate scattered medical knowledge, provide easily understandable risk explanations, treatment comparisons, and self-management advice tailored to users’ specific contexts, effectively compensating for the lack of timeliness and personalization in traditional health education approaches [5]. Especially in the field of chronic disease management, AI technology has shown multidimensional application potential in assisting medication guidance, symptom monitoring, and lifestyle intervention, providing a new technological pathway to improve patients’ health literacy and decision-making capacity [6].

However, the application of generative AI among older adults with multimorbidity still faces significant challenges. On the one hand, this population presents obvious characteristics of the digital divide, including insufficient digital access, limited technical operational ability, and low eHealth literacy, which prevent them from fully benefiting from the health dividends brought by AI technology [7]. On the other hand, existing generative AI products have obvious shortcomings in age-friendly design, such as complex interface interaction, information presentation that does not match the cognitive characteristics of older users, and a lack of in-depth understanding of the complex context of multimorbidity [8]. More importantly, when older patients translate AI recommendations into actual health decisions, they often encounter practical dilemmas including difficulties in judging information credibility, conflicts with existing medical regimens, and a lack of professional verification channels, making it difficult to effectively convert the potential value of AI into actual health benefits [9].

Current research on the experiences and mechanisms of generative AI use among older adults with multimorbidity remains significantly insufficient. Most existing literature focuses on younger populations or patients with a single chronic disease [10], and empirical studies targeting the complex context of multimorbidity are scarce, especially in-depth qualitative explorations based on theoretical frameworks. How do older patients perceive and respond to the benefits and risks of AI-assisted health decision-making in the context of changing cognitive function, multiple disease burdens, and evolving social support networks? What personal and social contextual factors moderate their technology acceptance process? Existing studies have not yet provided systematic answers. This knowledge gap restricts the design optimization and precise promotion of age-friendly AI health services, and also leaves healthcare practitioners without evidence-based guidance when advising older patients on the appropriate use of AI tools. Therefore, an in-depth understanding of the real experiences of this special group “dialoguing with algorithms” is urgently needed to promote the safe and effective application of generative AI in the management of chronic diseases among older adults.

### Theoretical framework

This study initially adopted the classic Technology Acceptance Model (TAM) [11–12] as a baseline analytical lens to interpret older adults’ perceived usefulness and perceived ease of use of generative artificial intelligence for complex health decision-making. However, the original Technology Acceptance Model cannot fully account for two core phenomena emerging from the interview data: the central role of personal and social resource mobilization, and the non-linear, dynamic usage trajectories that include trial, interruption, resumption and permanent abandonment among multimorbid older adults.

To address this limitation, we integrated the Senior Technology Acceptance Model (STAM) put forward by Chen and Chan in 2014 [13]. This age-sensitive extended theoretical framework is grounded in the Technology Acceptance Model and the Unified Theory of Acceptance and Use of Technology. The Senior Technology Acceptance Model supplements the original Technology Acceptance Model by introducing multiple ageing-specific capacity constructs, including cognitive ability, physical functioning, self-reported health, social relationships, and attitude to ageing. This model also redefines facilitating conditions as multi-layered personal and social support resources, a conceptualization that precisely aligns with the resource mobilization theme extracted from the present research. According to the Senior Technology Acceptance Model, there is a clear link between an individual’s resource mobilization and construction. First, personal resource mobilization covers dimensions such as electronic health literacy and self-assessment of cognitive and physical capacity. It corresponds to the age-related health and ability characteristics defined by the Senior Technology Acceptance Model, and serves as stable antecedents that directly shape perceived ease of use while moderating the association between perceived usefulness and sustained technology use. Second, social resource mobilization consists of family intergenerational guidance, professional counselling from community clinical staff and peer support among patients. It forms the core component of facilitating conditions, alleviating technology anxiety and lowering operational barriers to enhance perceived ease of use.

**Fig. 1:**
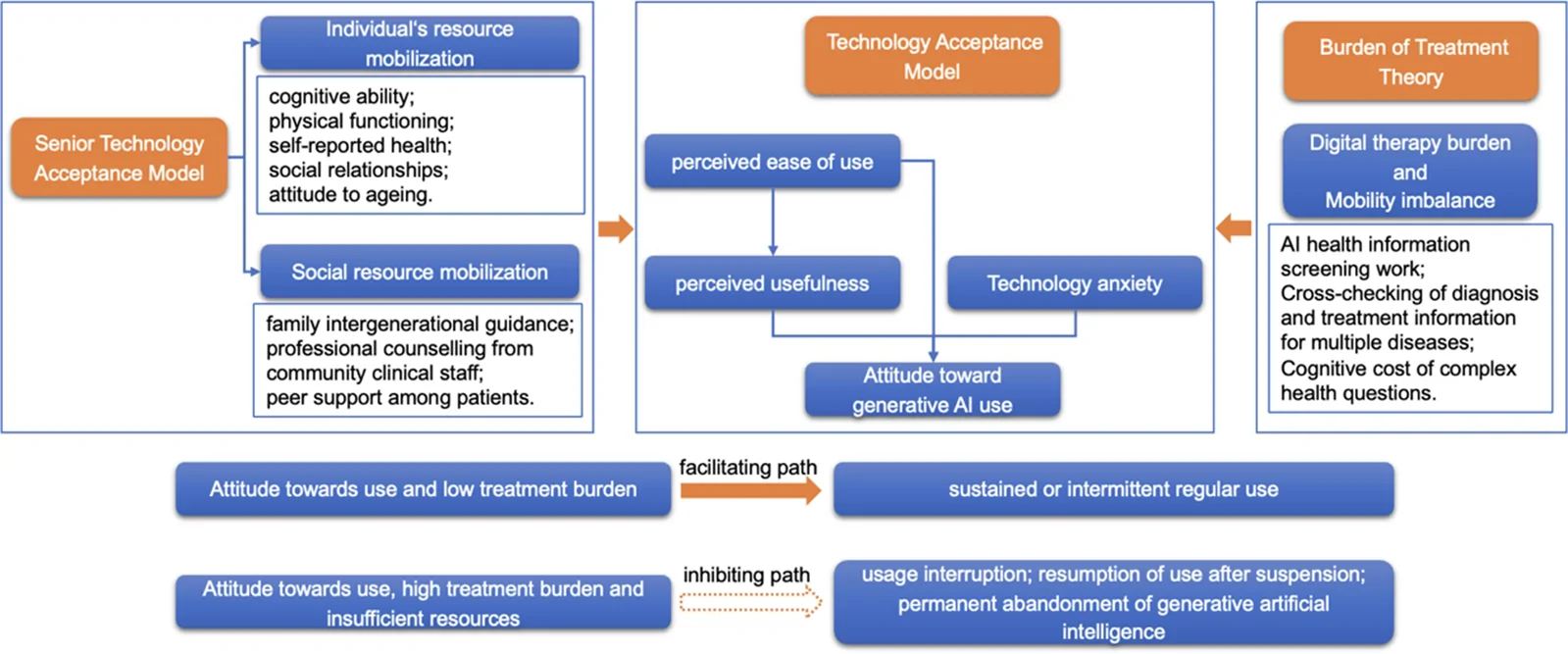
Overview of the integrated theoretical framework

Even after incorporating the Senior Technology Acceptance Model, the analytical system still lacks theoretical support to explain a critical contradiction observed by researchers: many older adults fully acknowledged the high practical value of generative artificial intelligence but still suspended or completely stopped using such tools. To fill this explanatory gap, we further introduced the Burden of Treatment Theory (BOTT) proposed by May and colleagues in 2014 into our integrated analytical framework [14]. The Burden of Treatment Theory centers on the imbalance between health management workload and an individual’s overall capacity for action for patients living with multiple chronic conditions. Within the context of this study, operating generative artificial intelligence, screening health information output by such tools, and verifying AI-generated medical suggestions against professional clinical opinions create additional digital care workload for elderly participants. When the cognitive, temporal and social resource costs brought by generative artificial intelligence outweigh an older adult’s available capacity, an inhibiting path will take shape, resulting in interrupted or permanent disengagement from AI tools. This underlying mechanism will provide a complete theoretical explanation for the non-linear usage trajectories captured in our interview data. Figure 1 provides an overview of the integrated theoretical framework.

### Objectives

To the best of our knowledge, no qualitative studies to date have applied the Technology Acceptance Model to analyze the experiences of older adults with multimorbidity using generative AI to address complex health decisions.

Applying this model to this specific patient population allows us to explore the following research questions:

1. What is the perceived usefulness of generative AI for health decision-making among older adults with multimorbidity, and what factors facilitate or constrain their perceived ease of use?
2. What personal resources and social resources can patients mobilize to cope with challenges in technology use?
3. How do perceived usefulness and perceived ease of shape their actual usage behavior and health decision-making quality through facilitating paths and/or inhibiting paths?

The findings of this study carry dual academic value. They can deepen the academic understanding of the psychological mechanisms underlying technology acceptance among older patients and contribute to the fields of health informatics and geriatric medicine. They can also provide empirical evidence for healthcare institutions and AI product designers to support the optimization of age-friendly intelligent health services, thereby promoting the effective application and popularization of generative AI in the management of chronic diseases among older adults.

## Methods

### Study design

An exploratory qualitative study design was adopted in this study. Through one-on-one semi-structured interviews, an in-depth understanding was obtained of the psychological experiences of older adults with multimorbidity when using generative AI to address complex health decisions. This design is particularly suitable for comprehensively and concisely summarizing participants’ experiences, enabling in-depth data analysis and describing the phenomenon within a specific context. This study was conducted in strict accordance with reporting guidelines for qualitative research, and findings were reported following the Consolidated Criteria for Reporting Qualitative Research (COREQ) checklist [15].

### Setting

This study was conducted at a community health service center in a provincial capital city in eastern China. The city enjoys a high level of economic and social development, ranking 8th among Chinese cities in terms of gross domestic product in 2024. The selected community health service center is located in the urban core district of the city. As a primary-level institution in the Chinese healthcare system, it mainly provides residents in its jurisdiction with medical treatment, prevention, health care, rehabilitation, and other health services, as well as free national basic public health services and contracted family doctor services. The community health service center employs approximately 150 medical and technical professionals, serves a total population of more than 30,000 people, of whom over 35% are aged 60 years and above, and provides more than 15,000 visits for older adults annually.

### Participants

From September to December 2025, 16 participants (P1–P16) were recruited from attendees at a community health service center in a provincial capital city in eastern China using purposive sampling and maximum variation sampling to ensure diversity in perspectives. Three key factors were considered in participant selection: (1) different age groups; (2) different disease combinations; and (3) different frequencies of AI use.

Inclusion criteria were: (1) aged 60 years or older; (2) diagnosed with two or more chronic diseases simultaneously, such as hypertension, diabetes, coronary heart disease, arthritis, etc.;(3) experience of using generative AI (e.g., ChatGPT, DeepSeek, Doubao, Kimi, etc.) to query health information within the past month; (4) currently facing or having previously experienced complex medical decision-making situations (e.g., decisions regarding surgery, medication regimens, specialist referral, etc.);(5) able to communicate in Chinese and clearly express their experiences.

Exclusion criteria were: (1) severe organic diseases; (2) severe neuropsychiatric disorders; (3) severe communication barriers.

### Sample size

Sample size was determined based on the assessment of data saturation during iterative data collection. Prior to participant recruitment, we set a pragmatic stopping rule: recruitment would cease if three consecutive interviews yielded no new core themes or distinct subthemes relevant to the study’s research questions. After each one-on-one interview, the two primary coders independently transcribed, coded, and drafted reflective memos of participants’ narratives. The full research team convened weekly to collate emergent codes and compare thematic content across all completed interviews to track the emergence of novel insights. Novel thematic content declined markedly from the 12th interview onward; no new core subthemes were identified across the 14th, 15th, and 16th interviews. Although saturation is not an absolute, definitive endpoint in qualitative inquiry, the consistent absence of unaddressed perspectives indicated the dataset contained sufficient breadth and depth to answer the research questions. Recruitment was therefore concluded after 16 participants.

### Participant characteristics

A total of 16 older adults with multimorbidity participated in this study, including 7 males (43.8%) and 9 females (56.2%), aged 64 to 78 years. The characteristics of the participants are presented in Table 1.

**Table 1:** characteristics of the participants

ID	Age(years)	Gender	Educational level	Types of chronic diseases	Living alone status	Frequency of AI use
P1	67	Female	Junior high school	Chronic heart failure complicated with anemia	Yes	5–6 times per week
P2	68	Male	Junior high school	Hypertension complicated with coronary heart disease	No	7 times per week or more
P3	64	Female	University	Post-operative breast cancer complicated with hypothyroidism	No	7 times per week or more
P4	69	Female	Junior high school	Hypertension complicated with diabetes	No	3–4 times per week
P5	70	Female	Senior high school	Multimorbidity	No	5–6 times per week
P6	76	Male	Primary school	Sequelae of cerebral infarction complicated with hyperlipidemia	Yes	1–2 times per week
P7	72	Female	Senior high school	Diabetes complicated with arthritis	No	3–4 times per week
P8	74	Male	Primary school	Chronic gastritis complicated with benign prostatic hyperplasia	No	1–2 times per week
P9	73	Male	Junior high school	Coronary heart disease complicated with chronic kidney disease	Yes	1–2 times per week
P10	72	Female	Junior high school	Coronary heart disease complicated with osteoporosis	No	1–2 times per week
P11	71	Male	Senior high school	Unstable angina complicated with chronic obstructive pulmonary disease	No	3–4 times per week
P12	75	Male	Primary school	Glaucoma complicated with benign prostatic hyperplasia	No	5–6 times per week
P13	71	Female	Junior high school	Chronic obstructive pulmonary disease complicated with hypertension	Yes	3–4 times per week
P14	68	Female	Senior high school	Atrial fibrillation complicated with osteoporosis	No	5–6 times per week
P15	78	Male	Primary school	Early Parkinson’s disease complicated with cataract	Yes	1–2 times per week
P16	74	Female	Primary school	Rheumatoid arthritis complicated with hypertension	No	3–4 times per week

### Development of the interview guide

The interview guide was developed based on an integrated framework of the Technology Acceptance Model, Senior Technology Acceptance Model, and Burden of Treatment Theory, combined with a review of previous study [16] and the research team’s professional expertise. Key questions focused on participants’ experiences of using generative AI to address complex health decisions, such as: “When did you first use AI to consult health-related issues, and what circumstances prompted you to do so?” “In what specific health decision-making contexts do you choose to ask AI?” “Describe one most memorable experience of discussing health issues with AI. How did you ask your questions, and how did AI respond?” and “Did you encounter any difficulties or frustrations when using AI to search for health information?” (Supplementary Materials). The interview guide was first pilot-tested by one researcher (Qin Lin) with two older adults with multimorbidity to evaluate the understandability and relevance of the questions and refine the interview procedure. Based on feedback, minor revisions and adjustments were made to improve the clarity of questions and participants’ comfort. Once finalized, the interview guide was applied consistently in all subsequent interviews. Interviews were conducted in the health education classroom of the community health service center, a quiet and private room. Interview duration ranged from 36 to 69 min, with an average of 46 min.

### Data collection

Data were collected through face‑to‑face, one‑on‑one semi‑structured interviews. All interviews were conducted in Mandarin and the local dialect (participants’ native language). The interview guide was developed in Chinese and subsequently translated into English under the guidance of native English speakers for reporting purposes. Before the interview, participants’ general sociodemographic information was collected, including age, sex, educational level, number of chronic diseases, living alone status, and frequency of AI use. After obtaining informed consent, all interviews were digitally recorded using portable devices (mobile phone or Microsoft Teams). During interviews, participants were encouraged to actively and freely share their views and experiences. Discussions centered on the experiences of older adults with multimorbidity in using generative AI for complex health decisions, as well as factors influencing their use of generative AI. All interviews were conducted by two researchers (Qin Lin and Pei Chen), who have extensive clinical practice and research experience in the management of older adults with chronic diseases and received training in qualitative research methods. Immediately after each interview, the research team held reflective discussions and summaries to review the interview process, document findings in a timely manner, and address challenges encountered during the process.

### Context of generative AI utilization

To strengthen methodological transparency and facilitate interpretive analysis of themes surrounding AI-assisted health decision-making, this subsection delineates contextual features of participants’ generative artificial intelligence engagement. All interviewees reported prior experience with at least one of three mainstream domestic generative artificial intelligence platforms: Doubao, Kimi, and DeepSeek.

In terms of payment modes, most participants only accessed free basic versions with word and functional restrictions, while a small proportion of seniors purchased low-cost monthly subscription packages to unlock complete health inquiry functions. Two distinct operation modes were identified across the sample: independent self-use by participants with intact cognitive and mobile operation ability, and mediated assisted use where family members or community clinical staff helped input health information, upload inspection reports and interpret AI-generated health feedback for older adults with impaired vision or memory.

The core health-related tasks and inquiry prompts submitted by participants centered on multimorbidity coordination, polypharmacy conflict screening, symptom discrimination, long-term disease management planning and interpretation of physical examination results, all closely linked to complex health decision-making for older adults living with multiple chronic conditions. Notably, no standardized, systematic professional guidance on generative artificial intelligence health consultation was provided by hospitals or community health service stations before participants began using these three tools. All operational skills and inquiry logic were acquired through self-exploration or informal one-on-one tutoring from relatives and peer elderly friends.

### Data analysis

All interview records were transcribed verbatim. All audio recordings were transcribed within 24 h using the iFlytek (China) online transcription service, followed by manual verification by two researchers (Qin Lin and Pei Chen). The transcribed interview records were imported into NVivo 15.0 software for data management. Data analysis was performed in accordance with reflexive thematic analysis, which has been validated to capture and interpret the meanings and content constructed by participants through repeated engagement with the data [17]. Reflexive thematic analysis facilitates the exploration of study themes and reveals the interactions between individual experiences and social influences. This methodology not only supports the theoretical framework and objectives of the study but also provides a robust approach to uncovering the psychological experiences of older adults with multimorbidity using generative AI for complex health decisions. Reflexive thematic analysis was conducted through six phases: (1) familiarization with the data; (2) generation of initial codes; (3) development of initial themes; (4) development and review of themes; (5) refinement and definition of themes; and (6) production of the research report. To ensure transparency of the analytical process, codes with similar meanings were grouped and further abstracted. Two researchers read the interview transcripts verbatim, wrote reflective notes, captured meaningful content at both semantic and latent levels, generated initial codes, and categorized codes into themes of consistent meaning. All authors jointly reviewed, refined, and defined the themes, and final themes were established through group discussion. In total, 4 main themes and 13 subthemes were derived from the reflexive thematic analysis (Supplementary Materials).

In this study, the Technology Acceptance Model, the Senior Technology Acceptance Model and the Burden of Treatment Theory were adopted as a posteriori interpretive tool rather than a priori rigid coding templates. All thematic extraction strictly followed the inductive logic of reflexive thematic analysis. During open coding, all initial codes and subthemes were purely induced from participants’ original interview narratives without forced matching with model dimensions. Multiple standardized strategies were implemented to avoid theory-driven overcoding caused by pre-set theoretical presuppositions. First, two independent researchers completed open coding separately without access to the integrated theoretical framework in the coding stage. Second, member checking was conducted with two interviewed older adults; we fed back preliminary thematic summaries to participants to verify whether the extracted themes conformed to their real experiences, rather than biased interpretations derived from theoretical assumptions. Third, a complete audit trail was maintained throughout data analysis. The research team held regular reflective group discussions to sort out all mismatches between raw interview content and the three theoretical models, and unified thematic definitions through collective negotiation to eliminate the bias of imposing theoretical logic onto original data. Only after all inductive subthemes were fully finalized did the research team carry out deductive matching between empirical themes and the three integrated theoretical models for result interpretation.

### Rigor

This study consistent with reflexive thematic analysis tenets. All team members have nursing and geriatric training, which shaped pre-existing interpretive assumptions about older multimorbid adults’ generative AI use. To mitigate disciplinary bias, all researchers kept reflective journals throughout data collection and coding. Two researchers independently completed inductive open coding. Discrepancies were not forced into consensus; competing interpretations were retained and cross-referenced against raw interview transcripts to enrich thematic nuance. Regular team reflexive meetings were held to examine biased inferences and iteratively refine themes from multiple interpretive perspectives. Two rounds of member checking were conducted with two participants exhibiting distinct non-linear AI usage trajectories. Instead of only verifying transcript accuracy, we shared thematic summaries and asked respondents to judge whether interpretations matched their lived experiences, identify missing experiential dimensions of resource mobilization, and flag misinterpretations. All feedback was documented in the audit trail and used to revise subthemes. Though limited to two participants, their contrasting usage paths effectively tempered disciplinary interpretive bias. A complete audit trail was maintained to archive reflective memos, divergent coding notes and iterative thematic revisions, transparently recording the reflexive analytical process.

### Ethical considerations

This study was approved by the hospital ethics committee (Approval No. 2025‑KJ(CDSJ)‑024‑01‑P) and conducted strictly in accordance with the principles of the Declaration of Helsinki. All participants provided written informed consent and were clearly informed that their participation was entirely voluntary and that they had the right to withdraw at any time without any adverse consequences. These details were reconfirmed by the researchers before the start of each interview. To protect participants’ privacy, anonymous coding of identifying information and data encryption were applied throughout the study. All research data were stored in a secure location on a password‑protected laptop computer with access restricted only to the researcher (Qin Lin). The researchers were fully prepared to maintain participants’ emotional stability during the discussions. If a participant experienced emotional distress or adverse reactions, the interview would be paused or terminated. Psychological counseling referral services from a mental health hospital were available if needed; however, none of the participants experienced such situations.

## Results

**Fig. 2:**
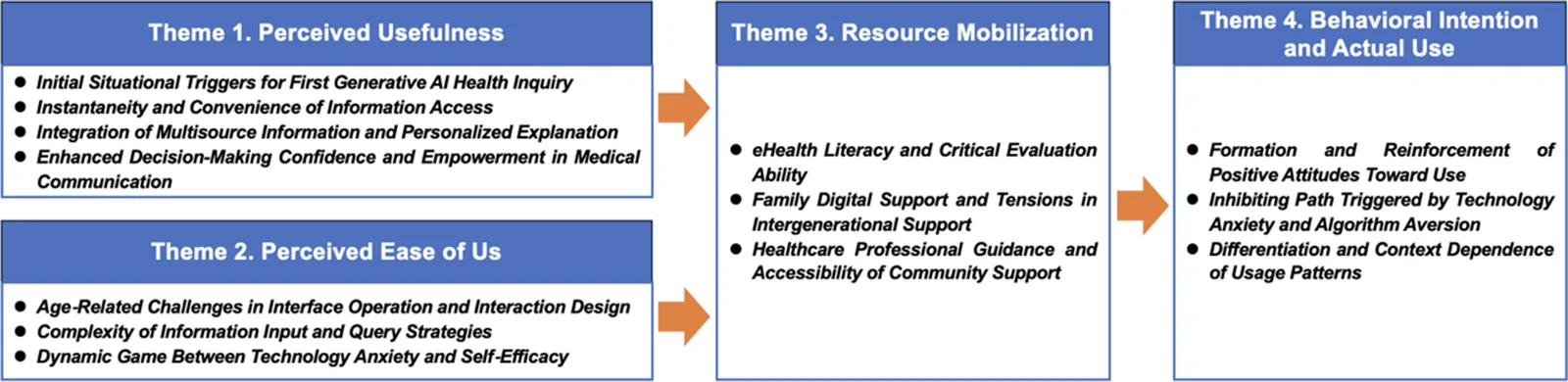
Main themes and sub-themes

A total of 4 main themes and 13 subthemes were derived from the reflexive thematic analysis. These themes were categorized into four categories, namely perceived usefulness, perceived ease of use, resource mobilization, and behavioral intention and actual use (Fig. 2). There were significant differences in the presentation rates of the above themes and subthemes among the 16 participants: some themes were almost universal, while others were only mentioned by a subset of participants.

### Perceived usefulness: AI as a digital advisor for health decision-making

During their interactions with generative AI, older adults with multimorbidity generally recognized its practical value in supporting health decision-making. Such perceived usefulness was mainly reflected in four dimensions: initial situational triggers prompting AI consultation, convenient access to information, integrated support for multimorbidity management, and enhanced decision-making confidence. However, patients’ perceptions of AI usefulness were not uniform, but were moderated by the complexity of their conditions, previous medical experiences, and level of digital literacy. Notably, even when perceiving AI as useful, patients maintained a rational positioning of AI as an adjunct rather than a replacement, viewing it as a supplement to traditional medical consultation rather than a substitute.

#### Initial situational triggers for first generative AI health inquiry

Multimorbid older adults initially turned to generative artificial intelligence to resolve practical barriers embedded within conventional medical services. Major triggering scenarios included inconsistent advice issued by clinicians across different departments for coexisting chronic illnesses, lack of immediate family support to sort out complex medication information, and urgent physical discomfort occurring after clinic opening hours, when primary care physicians are unavailable for timely consultation. These persistent healthcare inconveniences motivated participants to seek auxiliary health guidance from AI tools, laying the foundation for their subsequent recognition of AI’s practical value in health management.

- “I have high blood pressure plus diabetes and joint pain; each doctor only talks about one illness, their suggestions conflict. I can’t make sense of it, so I came to ask AI to sort all the medicines together.” (P5).
- “I live alone, my children only come back once a month. I have two chronic illnesses and cannot sort out all the doctor’s advice by myself, so I turned to AI to untangle my complicated medication plan at home.” (P13).
- “It’s troublesome to queue up at the hospital just for a minor discomfort at night. I ask AI first to figure out whether I need to rush to hospital immediately.” (P9).

#### Instantaneity and convenience of information access

Almost all participants emphasized the unique advantages of generative AI in breaking temporal and spatial constraints and providing rapid responses to health concerns. For older patients living with multiple chronic conditions who frequently encounter sudden symptoms or doubts about medications, the 24‑hour availability of AI filled the temporal and spatial gaps in traditional healthcare, allowing them to receive immediate psychological reassurance and information support outside of outpatient hours. This instantaneity not only alleviated disease-related anxiety but also endowed patients with a sense of agency to cope autonomously in urgent situations.

- “I suddenly felt palpitations after eleven o’clock at night and wondered if it was a side effect of the new antihypertensive drug. My son was already asleep, and the hospital was far away, so I tried asking AI. It immediately told me the possible causes and suggested I monitor my blood pressure. That put my mind at ease; I didn’t have to wait anxiously until dawn.” (P2).
- “In the past, I had to go to the community hospital and queue up even for a small problem. Now I can ask on my phone at home and know what to do in a few minutes. Especially after the COVID-19 pandemic, I got used to it and can’t live without it now; it’s so convenient.” (P7).
- “My eyes are bad, and I struggle to read the small words on the instructions. Now I just ask AI by voice; it reads aloud to me and explains what those medical terms mean. That’s much better than reading books by myself.” (P12).

#### Integration of multisource information and personalized explanation

Participants generally reported that generative AI could integrate fragmented information scattered across different specialties and drug instructions into coherent, personalized health recommendations. For older patients taking multiple medications and needing to coordinate prescriptions from different departments, this integration function was particularly valuable. AI not only provided standardized medical knowledge but also generated targeted analyses based on patients’ specific symptom combinations and medication lists, helping them understand interactions between different diseases and potential conflicts among treatment regimens.

- “I have hypertension, diabetes, and gout. Sometimes I can’t figure out if the medicines prescribed by the three doctors can be taken together. AI checked for me and said several drugs should be taken two hours apart, and explained why. It clarified many things I didn’t understand before.” (P5).
- “After the operation, the doctor told me to watch my diet, but there were conflicting opinions about what I could or couldn’t eat. I asked AI, and based on my diabetes and kidney disease, it listed a detailed diet plan and explained why I need to control protein. That’s much more reliable than searching online by myself.” (P9).

#### Enhanced decision-making confidence and empowerment in medical communication

Some participants reported that their self-efficacy in facing medical decisions improved significantly after using AI. By understanding disease knowledge, pros and cons of treatment options, and alternatives in advance, patients could communicate more effectively with physicians within limited outpatient time and even proactively raise questions based on AI searches. Such “prepared care-seeking” not only strengthened patients’ initiative but also alleviated feelings of powerlessness and anxiety when facing the authoritative medical system.

- “In the past, specialists would dismiss me in five minutes, and I was so nervous that I forgot what to ask. Now I first ask AI, write down unclear terms and possible side effects, and ask the doctor one by one during the visit. I feel like I go with my head, not just following orders.” (P3).
- “The doctor suggested a cardiac stent, but I was unsure. I asked AI about the pros and cons of the surgery and how long recovery would take. Once I had a clear understanding, I could follow the doctor’s explanation and voice my concerns. Finally, we agreed on a more suitable plan for me.” (P1).
- “AI told me there are two ways to take this new medicine: once a day or once a week, similar efficacy but different side effects. I took this information to my doctor, and we discussed which one to choose. I felt the doctor respected my choice more, instead of just telling me ‘take it this way’.” (P16).

### Perceived ease of use: threshold effects in crossing the digital divide

Older adults with multimorbidity exhibited substantial individual differences and context dependence in their perceived ease of use of generative AI. Although some participants could operate AI tools relatively smoothly, many patients encountered multiple operational barriers in the initial stage, including difficulties in interface navigation, input limitations, and challenges in result interpretation. Such “threshold effects” stemmed not only from physiological decline (e.g., reduced vision, impaired finger dexterity) but also from digital literacy levels and technology anxiety. Notably, perceived ease of use was not static but evolved dynamically with accumulated usage experience, age-friendly functional adaptation, and social support intervention, forming a continuum from technological fear to proficient operation.

#### Age‑related challenges in interface operation and interaction design

Most participants mentioned that current generative AI products had not adequately considered the physiological characteristics of older users in interface design, with problems such as excessively small fonts, dense buttons, and unclear feedback. Although voice interaction lowered the input threshold, speech recognition accuracy was affected by dialect accents, speech rate, and background noise, leading to repeated input failures and increased frustration. Some patients developed strong self‑doubt due to inability to complete basic operations independently and even abandoned attempts at an early stage.

- “The words are too small. I have to lean very close even with reading glasses, and sometimes I press the wrong button and don’t know how to go back. It makes me sweat with anxiety.” (P4).
- “I don’t speak standard Mandarin; I have a local accent. AI always mishears my questions. I asked ‘how to take aspirin’, but it heard ‘amoxicillin’ and gave an irrelevant answer. I can’t type, so I just feel helpless.” (P6).
- “The first time I used it, the interface kept jumping. I didn’t know where I clicked, and suddenly a lot of confusing things popped up. I just closed it in fear, thinking I broke the phone.” (P10).

#### Complexity of information input and query strategies

Even with basic operational skills, effectively asking questions to AI remained a major challenge for older patients. Participants generally lacked the ability to translate complex health situations into AI‑understandable instructions, often receiving inaccurate responses due to overly vague questions or insufficient key details. Some patients reported that the interaction logic of AI differed significantly from daily interpersonal communication and required learning specific machine language, and such cognitive conversion cost substantially increased usage burden.

- “I didn’t know how to ask it. I just said ‘I feel unwell’, and it asked where and how, but I couldn’t explain clearly. The final answer was too general and useless.” (P1).
- “I asked ‘what should I eat for my illness’, and it listed a lot. But I have diabetes and can’t eat some things. I forgot to tell it, so I had to ask again. It would be better if it could ask me about other diseases proactively, like a doctor does.” (P8).
- “Sometimes its answers are too long, with dense words. I forget the first half by the time I get to the middle and don’t know the key points. It would be better if it could tell me the three most important points first, like a doctor, and then I could ask for details.” (P13).

#### Dynamic game between technology anxiety and self‑efficacy

Perceived ease of use was related not only to objective operational difficulty but also to subjective psychological experience. Some participants showed obvious technology anxiety at the initial stage, worrying about misoperation causing device damage, privacy leakage, or incorrect medical information. However, with successful usage experience and age‑friendly auxiliary functions (e.g., preset quick commands by children, large‑font interfaces), some patients gradually improved their self‑efficacy and formed a positive cycle. Conversely, repeated early failures without support might solidify the negative self‑perception of “I cannot learn”, leading to permanent abandonment.

- “I’m always afraid of pressing the wrong thing, losing money in my phone, or having my medical records stolen by bad people. I feel nervous every time I use it, and my hands shake.” (P15).
- “My son set up a shortcut for me. One click and I can ask questions by voice without searching around. After several successful uses, I now dare to click by myself and am not as scared as before.” (P3).
- “I tried three times and failed. I thought I was too stupid and couldn’t keep up with the times. Later my granddaughter taught me patiently step by step. Now I’m slow but can search by myself. I feel I’m not senile yet.” (P9).

#### Resource mobilization: synergy between personal and social support systems

Successful use of generative AI among older adults with multimorbidity rarely relied on independent individual effort, but required the mobilization of diverse personal resources and social support. This theme revealed the interactive nature of the technology acceptance process: personal resources (e.g., eHealth literacy, technological self-efficacy, cognitive ability) formed the basic capacity for use, while social resources (e.g., family digital support, peer assistance, professional guidance from healthcare providers) provided critical backing. Notably, resource mobilization was not a simple superposition of resources but involved complex interactive effects: some social support might enhance personal efficacy, whereas excessive hands‑on help could weaken patients’ motivation for independent learning.

#### eHealth literacy and critical evaluation ability

Participants with higher eHealth literacy demonstrated stronger information screening ability and risk awareness, able to identify limitations of AI‑generated content, actively cross‑verify key information, and rationally evaluate the applicability of AI recommendations. In contrast, patients with lower health literacy tended to adopt extreme attitudes of complete trust or total distrust toward AI outputs, either blindly following inappropriate advice or missing useful information due to excessive skepticism. Such ability differences significantly influenced the safety and effectiveness of AI use.

- “AI said I could take this medicine, but I remembered my doctor warned me to use it carefully because of my poor kidney function. So I checked the drug instructions again and consulted the community doctor. I only dared to take it after confirmation. I can’t just believe everything it says; I have to be careful.” (P11).
- “I used to be a nurse. Although retired, I can read laboratory sheets and understand some medical terms, so I can roughly judge whether AI recommendations are reliable. People who know nothing might be misled.” (P7).
- “I read online that AI sometimes ‘talks nonsense’, so I will never rely on it alone for important questions; I must ask a doctor. But it explains clearly what some tests are for, and I think such general questions can be trusted.” (P5).
- “I cannot understand those professional medical words AI writes down. Once I fully followed its advice to adjust my blood pressure pills without asking anyone, and felt dizzy afterward. Now I think all AI medical suggestions are untrustworthy and I never open it again.” (P8).

#### Family digital support and tensions in intergenerational support

Adult children were the primary source of support for older patients using AI, with support ranging from full hands‑on help to guided instruction. Full hands‑on help solved immediate problems but might increase patient dependence and reduce self‑efficacy. Patient, guided instruction, although less efficient initially, helped patients gradually build independent use skills. In addition, differences in health beliefs and technology use habits between generations occasionally caused conflicts, such as children over intervening in patients’ AI queries or patients developing resistance to digital instruction from children.

- “My son just takes my phone and searches for me every time, then tells me the result. I know he’s good to me, but I still haven’t learned how to do it myself. I have to ask him next time; I feel useless.” (P2).
- “When my daughter teaches me, she lets me operate by myself while watching and correcting my mistakes. Although I learn slowly, I can now do basic things without bothering her every time. I feel much more secure.” (P12).
- “My granddaughter was impatient when teaching me, complained I was slow, and said ‘Forget it, I’ll check for you’. I felt terrible, like a burden. Later I practiced secretly because I didn’t want to keep asking her.” (P14).

#### Healthcare professional guidance and accessibility of community support

Healthcare providers in community health service centers played dual roles as gatekeepers and guides in older patients’ AI use. Some participants mentioned that positive attitudes from healthcare providers significantly enhanced their use confidence, whereas negative or skeptical attitudes intensified patients’ technology anxiety. In addition, digital literacy training organized by the community and experience sharing among patients provided important supplements to resource mobilization, yet the coverage and accessibility of such formal support resources remained insufficient.

- “My family doctor knew I used AI to search for information. Instead of criticizing me, he told me which apps were reliable and warned me not to reveal personal privacy. With a doctor’s approval, I feel more confident using it.” (P16).
- “I asked the community doctor about an examination recommended by AI. He was impatient and said ‘Do you believe it or me?’ I felt like I had done something wrong and didn’t dare mention AI in front of him again.” (P6).
- “The community held several smartphone training sessions teaching us how to use health codes and register, but not how to use AI for health questions. A few of us patients teach each other; whoever finds a good function tells everyone else. But sometimes we’re afraid we might teach the wrong thing.” (P10).

### Behavioral intention and actual use: evolutionary trajectory from trial adoption to sustained dependence

The generative AI usage behavior of older adults with multimorbidity exhibited dynamic evolutionary characteristics. Its trajectory was not a linear process of acceptance and continuous use, but a complex journey involving attempts, interruptions, resumption, or permanent abandonment. This process was profoundly influenced by the interaction between perceived usefulness and perceived ease of use, and underwent turning points triggered by key contextual factors. Some patients gradually integrated AI into their daily health management as a routine tool, forming sustained dependence; others, however, discontinued use due to technical barriers, trust crises, or the emergence of alternative solutions, returning to traditional information-seeking patterns.

#### Formation and reinforcement of positive attitudes toward use

When patients simultaneously experienced high usefulness and acceptable ease of use, they developed positive attitudes toward AI use, manifested as proactively exploring AI functions, recommending AI to others, and prioritizing AI in various health contexts. The formation of such attitudes was often associated with key successful events, for example, when an AI recommendation helped a patient promptly identify an adverse drug reaction, or when AI provided effective psychological reassurance while waiting for emergency care. Once formed, positive attitudes were highly stable; even in the face of occasional technical failures, patients tended to attribute them to temporary issues rather than denying the value of AI.

- “Since the time AI helped me discover a potential conflict between two medications I was taking, and I told my doctor to adjust the regimen, I have felt this tool is really useful. Now I recommend it to everyone I know, and several classmates from my senior university have started using it too.” (P3).
- “Every morning when I get up, I first ask AI about the weather and suitable exercises for the day, then check my blood glucose control target. It has become a habit, as natural as brushing my teeth and washing my face.” (P7).
- “Although sometimes its answers are not very accurate, it has helped me a lot most of the time. I think we shouldn’t throw the baby out with the bathwater, just like doctors don’t make correct diagnoses every time, but we still go to see them.” (P9).

#### Inhibiting path triggered by technology anxiety and algorithm aversion

Some patients entered an inhibiting path after initial attempts due to increased technology anxiety or experiences of algorithmic failure. Algorithm aversion was manifested as distrust in AI decisions and criticism that AI lacked humanistic care. If such negative emotions were not promptly intervened, they might quickly solidify into the belief that “older adults should not rely on these fancy things,” leading to discontinued use or even technological resistance. Notably, the formation of the inhibiting path often had a cascading effect: a single negative experience might spread through social networks, influencing the technology acceptance of surrounding older adults.

- “Once AI said my symptoms might be cancer, which scared me so much that I couldn’t sleep all night. I went to the hospital for an examination the next day, and it turned out to be nothing. I think this tool is too unreliable; I dare not use it again. I’d rather ask doctors honestly.” (P1).
- “My wife accidentally clicked on an advertisement once and downloaded a bunch of messy software, which crashed the phone. We were afraid of further problems, so we deleted that AI. We thought it was safer, better to not know than to take risks.” (P15).

#### Differentiation and context dependence of usage patterns

Even among patients who developed continuous use behaviors, their usage patterns showed significant differences: some patients evolved into deep integration users, using AI for complex decision analysis and long-term health monitoring; some maintained tool-based use, seeking help from AI only in specific contexts; others exhibited intermittent use characteristics, adjusting usage frequency according to fluctuations in their disease status. This differentiation reflected older patients’ strategic choices regarding AI functions, seeking a personalized balance between efficiency pursuit and risk avoidance, while also being moderated by the accessibility of medical resources.

- “My chronic diseases are stable now. I mainly use AI to check health preservation methods and dietary adjustments, but if I feel unwell or need to adjust my medications, I definitely go to the hospital and dare not make decisions on my own.” (P8).
- “Before each monthly follow-up, I organize my recent symptoms and blood pressure/blood glucose records, then ask AI how to communicate with my doctor and what questions I need to ask. It helps me sort out my thoughts, but I still follow the doctor’s final decision.” (P13).
- “I don’t think about using AI when I’m in good health, but last time I had an acute gout attack and was in terrible pain in the middle of the night. I asked AI how to handle it first aid and whether I needed to go to the emergency room. It suggested I apply ice, drink more water to observe, and go to the hospital the next day. I felt particularly need it at that time.” (P5).

## Discussion

### Theoretical integration: extended STAM as the unifying explanatory framework

This study adopts the Senior Technology Acceptance Model (STAM), a gerontological extension of the original Technology Acceptance Model (TAM), as the core integrative analytical framework to synthesize all emergent thematic findings. Traditional TAM centers on perceived usefulness and perceived ease of use as proximal predictors of technology adoption intention, yet it neglect age-specific individual and social constraints unique to multimorbid older adults. STAM remedies this gap by reserving perceived usefulness and perceived ease of use as core endogenous constructs while incorporating a set of critical external antecedent and conditioning variables that jointly shape the full intention-to-sustained-use pathway, which aligns perfectly with the four core themes derived from qualitative data. Specifically, eHealth literacy and chronic disease self-management capacity function as individual cognitive preconditions that jointly shape participants’ judgment of AI’s perceived usefulness and perceived ease of use [18]. Technology anxiety represents an age-related affective barrier that suppresses positive perceptions of generative AI, while intergenerational social support networks operate as external social resources buffering operational difficulties. The Burden of Treatment Theory (BOTT) further extends this STAM framework by incorporating healthcare access barriers and polypharmacy-driven decision burden as context-specific moderators, which adjust the magnitude of associations between PU/PEOU and long-term AI utilization. All four thematic dimensions extracted from interview data are systematically mapped onto components of this extended STAM framework:

1. Perceived Usefulness: Core endogenous TAM/STAM construct;
2. Perceived Ease of Use and Technology Anxiety: Age-specific usability and affective dimensions embedded within STAM;
3. eHealth Literacy and Critical Evaluation Ability: Individual cognitive external antecedents;
4. Social Support, Medical Access and Multimorbidity Treatment Burden: Social and healthcare contextual moderators integrated via BOTT.

This study establishes a hierarchical integrated model where STAM provides the primary structural backbone, and BOTT, eHealth literacy, chronic self-management, and social support act as supplementary context-specific extensions.

### Alignment with multivariable pre-engagement models of behavioral intention

Early TAM scholarship largely frames behavioral intention as a direct outcome of perceived usefulness and perceived ease of use, with limited attention to pre-existing psychosocial resources that shape uptake before formal tool exposure. Recent digital health research has shifted toward multivariable serial mediation models that position intention as a product of layered pre-engagement determinants beyond core TAM constructs. A quantitative study by Mazuz [19] identifies a sequential pathway of trauma literacy, self-efficacy, citizenship, adoption intention for crisis-focused mental health apps, proving that cognitive and social preconditions independently predict willingness to engage with digital interventions.

The present qualitative work extends this multivariable logic to the distinct population of multimorbid older adults engaging in generative AI health consultation. While Mazuz’s model centers on civilian trauma contexts and one-off pre-crisis app awareness, this study unpacks age-specific, chronic disease-related psychosocial antecedents including eHealth literacy, polypharmacy decision burden, and intergenerational support networks. These geriatric, disease-specific factors expand the scope of pre-engagement determinants, refining interpretations of behavioral intention for long-term, recurring AI health use rather than single emergency adoption decisions.

### Cultural and socioeconomic contextual boundaries of AI health engagement

Cultural norms rooted in Chinese family and medical culture substantially shape multimorbid older adults’ entire process of engaging with generative AI health tools, including operation, information judgment and clinical decision-making trade-offs [6]. First, strong intergenerational support norms form a family-dependent digital assistance model widely observed among participants. Many elderly respondents reported that they could not independently complete complex AI inquiry operations and would first send AI-generated medical advice to their adult children via WeChat for secondary screening; once children identified conflicts between algorithm suggestions and doctors’ previous instructions, seniors would directly abandon AI’s recommendations without further deliberation. Second, long-standing cultural deference to medical professional authority creates a hierarchical information evaluation logic. Multiple participants stated they automatically dismissed AI advice that contradicted face-to-face clinician diagnoses, even when the AI provided detailed medication comparison reasoning. Third, restrained, indirect communication styles reduce seniors’ willingness to independently question algorithmic flaws: most would rather rely on family mediation than directly judge AI’s potential bias or error on their own.

Beyond cultural factors, socioeconomic conditions may shape older adults’ access to generative AI and their self-confidence in utilizing such tools [20]. All participants were recruited from urban community health centers with accessible public digital training, stable smartphone ownership and affordable cellular data packages. In contrast, rural low-income elderly groups face multiple barriers: scarce offline digital literacy tutoring at village clinics, high replacement costs for smart devices and unstable mobile data signals. All participants in this study had stable access to urban community primary care services, whereas rural older adults may only receive limited grassroots medical support. Such uneven digital and medical resource access means the observed pathways of AI acceptance, critical evaluation and sustained use cannot be directly transferred to economically disadvantaged, digitally marginalized elderly populations in underdeveloped rural regions.

### Recognition of practical value and construction of rational boundaries

The present study found that the perceived usefulness of generative AI among older adults with multimorbidity was mainly reflected in three dimensions: instant access to information, integration of multisource information, and enhanced decision-making confidence. This finding is consistent with conclusions from studies on older adults in the United States using smart assistants for health information management [21], in which participants also highly valued the practical value of AI in supporting health information. However, unlike the priority effect of perceived ease of use reported in that study, patients with multimorbidity in the current study exhibited significantly higher sensitivity to perceived usefulness than to mere ease of use, due to their more complex decision-making contexts. This may stem from the urgency of information needs brought about by multimorbidity: when patients manage multiple chronic conditions and coordinate polypharmacy regimens, the utility of rapidly obtaining targeted advice far outweighs considerations of operational convenience.

In clinical practice, healthcare providers should fully recognize this population’s demand for high-quality health information. When recommending AI tools, priority should be given to emphasizing their supportive value in complex decision-making contexts, rather than focusing only on interface simplicity. Importantly, implementation strategies require cultural tailoring and resource-sensitive design. Interventions should align with Chinese intergenerational support norms and older adults’ inherent respect for clinical authority, reserving space for family members to participate in information screening and clarifying that AI only serves as an auxiliary tool for clinicians. Meanwhile, differentiated schemes should be formulated according to socioeconomic disparities: systematic online digital literacy training can be provided for urban seniors with stable digital access, while offline paper guides and village clinic-led voice AI terminals are more suitable for rural low-income elders limited by smartphone and data costs.

Notably, patients’ perceptions of AI usefulness in this study showed clear context dependence and characteristics of strategic choice. Participants did not unconditionally accept all AI outputs, but positioned AI as a supplement rather than a replacement for traditional medical consultation. This rational positioning reflects older patients’ agency and risk awareness in technology use. This echoes the phenomenon of authority skepticism found in a study on ethical considerations and attitudes toward AI in medical decision-making among older adults in Bangladesh [22], but the present study further reveals the health literacy foundation underlying such skepticism. The thematic data suggests a tentative pattern: participants with higher eHealth literacy appeared better able to judge the applicable scope of AI-generated advice, while those with limited literacy were prone to swing between full acceptance and complete rejection of algorithmic content. This experiential observation offers tentative practice insights. Targeted eHealth literacy training for older adults may support their capacity to assess AI outputs rationally, which could potentially lower the risks stemming from unreserved compliance or undue dismissal of valuable health guidance.

### Overcoming operational barriers and alleviating technology anxiety

The present study revealed significant threshold effects in perceived ease of use among older adults with multimorbidity, which were shaped by the interplay of multiple factors including physiological decline, weak digital literacy foundations, and technology anxiety. Unlike previous studies that emphasized technical design barriers [23], this study found that ease-of-use challenges stemmed not only from objective issues such as age-unfriendly interfaces but also from patients’ subjective psychological experiences. In this study, technology anxiety was manifested as excessive worry about the consequences of misoperation, fear of privacy leakage, and doubt about one’s own learning ability. Such anxiety could easily solidify into avoidance behavior after initial use failures. In clinical practice, simple technical training is often insufficient to overcome this deep-seated anxiety, and more supportive intervention strategies are needed. For example, patient guided instruction rather than full hands-on help from family members, positive recognition rather than outright denial from healthcare providers, and the design of a safe usage environment that allows for mistakes and backtracking can all help gradually rebuild patients’ self-efficacy. As a regular medical service venue for older patients, community health service centers should fully leverage their local advantages, set up AI use counseling windows, and provide one-on-one adaptive training.

The dynamic evolutionary characteristics of perceived ease of use identified in this study suggest that technology acceptance is a process rather than an event. Some participants gradually transitioned from relying on external support to independent operation through the accumulation of successful usage experiences, forming a positive trajectory of ability development. This finding is consistent with the perspective of self-efficacy theory, which holds that mastery experiences are the strongest source of improved self-efficacy [24]. However, interview narratives indicate some participants developed learned helplessness after repeated early operational setbacks and insufficient timely assistance, which was linked to subsequent discontinuation of AI tools. This contrast offers tentative insights into intervention timing: delivering adequate tailored support at the initial stage of AI exposure may correlate with sustained long-term engagement patterns, though longitudinal research is required to confirm this relationship. In addition, this study found that although voice interaction lowered the input threshold, it may instead increase frustration for some users due to factors such as dialect accents and background noise. This suggests that the age-friendly design of AI products needs to go beyond simple function addition, deeply consider the language characteristics and usage scenarios of the target users, and develop more inclusive interaction modes [25].

### Dual support from capacity foundation and support network

The present study emphasizes the synergistic mechanism of personal resources and social support in older patients’ AI use. As a salient personal resource, eHealth literacy appeared to correlate with participants’ basic operational proficiency, alongside their capacity to filter and critically assess AI-derived health information based on interview narratives [26]. This study found that participants with a medical background or higher educational level could more effectively integrate AI recommendations with their own disease experiences and doctor’s advice to form personalized health decisions. However, for the vast majority of older patients without a professional background, the quality of the social support system becomes a key factor in compensating for the lack of personal resources [27]. Family digital support in this study presented complex dual effects: the support methods of adult children, including full hands-on help and guided instruction, directly shaped patients’ learning outcomes and long-term use capabilities. While full hands-on help solved immediate problems, it deprived patients of practical opportunities and led to increased dependence; in contrast, patient guided instruction, although less efficient initially, helped patients gradually establish independent use capabilities. This finding has important guiding significance for family members: when assisting their elderly parents in using AI tools, they should restrain the urge to solve problems immediately and invest time in process-oriented guidance, which will bring more long-term benefits.

This study also revealed the insufficiency of formal support resources in older patients’ AI use. Although healthcare providers in community health service centers are regarded by patients as trustworthy sources of information verification, their attitudes toward AI technology are significantly differentiated. Some healthcare providers actively recognize and guide patients to use AI rationally, while others simply deny or avoid discussions. This inconsistency increases patients’ confusion and technology anxiety, and also reflects the ambiguity in the role positioning of primary medical personnel in the AI era. With the deepening penetration of generative AI in the health field, healthcare providers need to re-examine their professional boundaries, transforming from information monopolists to information curators and cultivators of critical thinking [28]. In addition, this study found that digital literacy training at the community level mostly focuses on basic operational skills, lacking specialized content targeting health information management and AI tool use. It is recommended to incorporate AI-assisted health decision-making into the community elderly education curriculum system, develop training modules suitable for the needs of patients with multimorbidity, and establish a peer support network among patients to promote experience sharing and peer support, forming a diversified social support ecosystem [29].

### Evolution of usage patterns and prospects for human-AI collaboration

The present study depicts the dynamic evolutionary trajectory of AI usage behavior among older adults with multimorbidity. This trajectory is not a linear process of continuous adoption, but a complex journey filled with attempts, interruptions, resumption, or permanent abandonment. The formation of a positive attitude toward use is often closely associated with key successful events, such as AI recommendations helping patients promptly identify drug interactions or providing effective reassurance during sudden nighttime symptoms [30]. These events not only verify the practical value of AI but also strengthen patients’ sense of self-efficacy and control. However, thematic observations from participant interviews point to a potentially disruptive association between negative algorithmic encounters and subsequent AI engagement patterns. Participants recounted that one prominent inaccurate AI recommendation, privacy leakage worries, or unpleasant ad click experiences appeared to shift their formerly favorable perceptions rapidly, which some participants linked to reduced usage or complete disengagement from generative AI health tools. This asymmetric effect suggests that in the high-risk field of healthcare, the accuracy and safety standards of AI systems must be far higher than those of general consumer-grade applications. In clinical practice, healthcare providers should proactively inquire about patients’ AI usage experiences, promptly clarify misunderstandings, correct misinformation, and provide supportive interventions when patients encounter negative experiences, preventing them from falling into a solidified path of technological resistance [31].

The phenomenon of differentiated usage patterns observed in this study reflects older patients’ strategic choices regarding AI functions. Deeply integrated users incorporate AI into their routine tools for daily health management; tool-based users seek help from AI only in specific contexts; and intermittent users adjust their usage frequency according to fluctuations in their disease status. This differentiation is not a reflection of varying abilities, but a result of individuals seeking a personalized balance between efficiency pursuit and risk avoidance. Clinical healthcare providers should respect this differentiation, avoid regarding continuous and high-frequency use as the only ideal goal, and instead help patients find the optimal usage mode that suits their disease characteristics, living situations, and cognitive styles [32]. In addition, this study found that the accessibility of medical resources has a significant moderating effect on usage patterns: when family doctor services are highly accessible and doctor-patient communication is sufficient, patients are more likely to use AI as a preparatory tool before medical visits; when specialist registration is difficult and the accessibility of the medical system is low, patients may over-rely on AI recommendations, increasing the risk of self-diagnosis and self-treatment. This suggests that while promoting AI-assisted health decision-making, it is imperative to simultaneously strengthen the construction of primary healthcare service capabilities, ensuring that patients can timely obtain verification and guidance from professionals when needed, and forming a health decision support system based on human-AI collaboration rather than human-AI replacement [33].

## Strengths and limitations

### Strengths

This study has several strengths. The most core strength is the detailed portrayal of the technology acceptance process among older adults with multimorbidity through in-depth qualitative research. This research method made it possible to explore context-specific factors influencing patients’ AI usage experiences, derive innovative research insights into the dynamic evolution mechanism of technology acceptance, and highlight the value of constructing an age-friendly support system. The study strictly followed the methodological requirements of reflexive thematic analysis, and ensured research rigor through strategies such as researcher triangulation, participant feedback, and audit trails, providing an empirical basis for understanding the application of the Technology Acceptance Model in the context of elderly chronic disease management.

### Limitations

This study also has certain limitations. First, the inclusion criterion restricted participants to those who had used generative AI for health inquiries within the past month, which creates a sampling limitation. This one-month time window excludes older adults who previously experimented with generative AI but permanently ceased all use before this period. Such long-term non-users represent a critical subgroup for unpacking the inhibiting path of technology acceptance, as their abandonment experiences stem from persistent technology anxiety, algorithm aversion, or unresolved operational barriers. By excluding this population, this may weaken the comprehensiveness of our findings regarding the full spectrum of negative usage trajectories and the long-term drivers of permanent technology abandonment among multimorbid older adults. Second, the study sample was only recruited from a community health service center in a provincial capital city in eastern China, and participants had a certain economic foundation and digital access conditions, which may limit the transferability of the study findings to elderly populations in rural or economically underdeveloped areas. In addition, although the researchers had professional backgrounds in nursing and geriatrics, enabling accurate qualitative interpretation of the research topic, this dual professional identity may have had potential impacts on the feedback results of the research subjects due to social desirability bias. To offset this bias: Before interviews, we emphasized all positive and negative experiences were equally valuable with no normative judgment; Interviewers received training to avoid clinical commentary and only use neutral open-ended prompts; Dual independent coding and team cross-review reduced interpretation bias from clinical mindsets. Fourth, consistent with the standard conventions of thematic analysis, participant feedback obtained via two rounds of member checking was only utilized to verify thematic resonance and correct any misinterpretations of participants’ lived experience. This feedback process cannot compensate for missing perspectives that participants may have withheld during interviews due to hesitation or self-censorship. Importantly, all thematic interpretations presented in this manuscript are inherently researcher-constructed, shaped by the research team’s nursing and gerontological backgrounds as well as the integrated theoretical framework adopted in this work, despite rigorous reflexive coding and team discussion procedures. Fifth, this research bears limitations concerning the construct validity of AI-assisted health decision-making quality. The analysis solely explores participants’ subjective perceptions, attitudes and self-reported experience regarding AI consultation. The dataset cannot generate objective evidence to confirm actual improvements or declines in real decision-making quality, medical decision safety, long-term disease control or other measurable clinical outcomes.

## Conclusion

This study analyzed the experiences of older adults with multimorbidity using generative AI to address complex health decisions within the theoretical framework of the Technology Acceptance Model (TAM), exploring the complex interactions between perceived usefulness, perceived ease of use, and contextual factors through the facilitating path and inhibiting path. The results indicate that AI usage behavior among this population is the outcome of the dynamic interaction of various factors against the backdrop of intertwined health information needs, technical capacity limitations, and social support networks. The findings of this study reveal that AI use among older adults with multimorbidity is not merely a technology adoption behavior, but an adaptive strategic choice made under the pressure of managing multiple chronic conditions. This also highlights a core necessity: the application of generative AI among older adults should be understood and promoted within an integrated framework that incorporates three dimensions—individual capabilities, interpersonal support, and the healthcare system. In clinical practice, healthcare providers should move beyond simple technology recommendations and instead focus on patients’ health literacy foundations, levels of technology anxiety, and the quality of their social support networks, providing differentiated guidance strategies. Meanwhile, educational guidance for family caregivers should be strengthened to promote their transformation from full hands-on helpers to guided instructors. Additionally, community health service centers should be encouraged to establish AI use counseling mechanisms, forming a collaborative support system involving hospitals, communities, and families.

## Supplementary Information

Below is the link to the electronic supplementary material.

Supplementary Material 1

## Data Availability

Ethical approval for this study from the REB did not permit the sharing of interview data in the public domain, as participants did not provide consent for such disclosure; accordingly, the data is unavailable.

## Abbreviation

AI: Artificial Intelligence
COREQ: Consolidated Criteria for Reporting Qualitative Research
ICT: Information and Communication Technology
TAM: Technology Acceptance Model
STAM: Senior Technology Acceptance Model
BOTT: Burden of Treatment Theory
