# Supplementary material for: Dialoguing with algorithms: experiences of generative AI use in navigating complex health decisions among older adults with multimorbidity: a qualitative study
Source: BMC Health Serv Res. 2026 Jul 23;26:1399. doi: 10.1186/s12913-026-15158-x (PMC13663120; doi:10.1186/s12913-026-15158-x)
Supplement: Supplementary file 1 [file 12913_2026_15158_MOESM1_ESM.docx]

**Supplemental Materials**

**Supplementary File:1**

**Consolidated Criteria for Reporting Qualitative Research (COREQ) 32-Item Checklist – Alignment with Manuscript**

This appendix presents how each of the 32 COREQ checklist items has been addressed in the manuscript *‘Dialoguing with algorithms: experiences of generative AI use in navigating complex health decisions among older adults with multimorbidity: A qualitative study’* The table below demonstrates compliance with each criterion in detail to ensure transparency and rigor in qualitative reporting.

| No. | Item | Guide Question / Description | How Addressed in the Manuscript |
| --- | --- | --- | --- |
| Domain 1: Research team and reﬂexivity | | | |
| *Personal Characteristics* | | | |
| 1 | Interviewer/facilitator | Which author(s) conducted the interview? | Interviews conducted by Qin Lin and Pei Chen under supervision of Minmin Jiang. |
| 2 | Credentials | What were the researcher’s credentials? | All researchers hold PhD or MSN. |
| 3 | Occupation | What was their occupation at the time of the study? | Five of them are medical school faculty, one is a doctoral student at the School of Nursing, and two are hospital nursing staff. |
| 4 | Gender | Was the researcher male or female? | Both male and female researchers participated. |
| 5 | Experience and training | What experience or training did the researcher have? | Researchers were trained and experienced in qualitative research. |
| *Relationship with participants* | | | |
| 6 | Relationship established | Was a relationship established prior to study commencement? | Initial contact was made through referrals from staff at the community health service center, followed by the informed consent procedure. |
| 7 | Participant knowledge of the interviewer | What did the participants know about the researcher? | Participants informed about study aims, study procedure, research team, and confidentiality. |
| 8 | Interviewer characteristics | What characteristics were reported about the interviewer? | Researchers maintained neutrality and reflexivity to minimize bias. |
| Domain 2: study design | | | |
| *Theoretical framework* | | | |
| 9 | Methodological orientation and theory | What methodological orientation underpinned the study? | The study adopted a qualitative descriptive design, and reflexive thematic analysis was applied for data analysis. |
| *Participant selection* | | | |
| 10 | Sampling | How were participants selected? | Participants were recruited using purposive sampling combined with maximum variation sampling from attendees of a community health service center in a provincial capital city of eastern China between September and December 2025. |
| 11 | Method of approach | How were participants approached? | Potential participants were first referred by staff from the community health service center. |
| 12 | Sample size | How many participants were in the study? | 16 participants. |
| 13 | Non-participation | How many refused or dropped out? | All invited participants completed interviews. |
| *Setting* | | | |
| 14 | Setting of data collection | Where was data collected? | At community health service center, universities, and online (iFlytek). |
| 15 | Presence of non-participants | Was anyone else present? | No, only interviewer and participant were present. |
| 16 | Description of sample | What are the key characteristics of participants? | Demographic characteristics shown in Table 1. |
| *Data collection* | | | |
| 17 | Interview guide | Were questions or prompts provided? | Semi-structured interview guide included as supplementary file. |
| 18 | Repeat interviews | Were repeat interviews conducted? | No, saturation achieved without repeats. |
| 19 | Audio/visual recording | Did the research use audio or visual recording? | all interviews were digitally recorded via portable devices (mobile phones or Microsoft Teams) for subsequent verbatim transcription. No visual/video recording was adopted in this study. |
| 20 | Field notes | Were field notes made? | Yes, contextual field notes taken to support interpretation. |
| 21 | Duration | What was interview duration? | Interviews lasted 36–69 minutes. |
| 22 | Data saturation | Was data saturation discussed? | Yes, Researchers set a stopping rule: recruitment would stop if three consecutive interviews yielded no new core or subthemes relevant to the research questions. Novel thematic content declined markedly starting from the 12th interview, and no new core subthemes emerged across the 14th, 15th, and 16th interviews. Therefore, recruitment was terminated after 16 participants. |
| 23 | Transcripts returned | Were transcripts returned for comment? | No full return; member checking performed after interviews. |
| Domain 3: analysis and ﬁndings | | | |
| *Data analysis* | | | |
| 24 | Number of data coders | How many data coders coded the data? | Two researchers (Yanping Niu and Jiaorong Zhao) independently completed dual coding of the data. |
| 25 | Description of coding tree | Did authors provide a description of the coding tree? | The study derived 4 main themes and 13 subthemes via reflexive thematic analysis, and the hierarchical structure of themes/subthemes (coding tree) was visualized in Figure 2. |
| 26 | Derivation of themes | Were themes identified in advance or derived from data? | Themes inductively derived from interview data. |
| 27 | Software | What software was used to manage the data? | NVivo 15.0 software. |
| 28 | Participant checking | Did participants provide feedback on findings? | Two rounds of member checking (participant checking) were carried out. The research purposively selected two participants with contrasting non-linear generative AI usage trajectories to collect feedback on study thematic findings. |
| *Reporting* | | | |
| 29 | Quotations presented | Were participant quotations used to illustrate themes? | Yes, representative quotes included with participant numbers. |
| 30 | Data and findings consistent | Was there consistency between data and findings? | Yes, results aligned with supporting quotes and analysis logic. |
| 31 | Clarity of major themes | Were major themes clearly presented? | Yes. Four clear main themes were systematically identified and fully presented throughout the manuscript. The four core themes included perceived usefulness, perceived ease of use, resource mobilization, and behavioral intention and actual use, with a total of 13 corresponding subthemes elaborated respectively. |
| 32 | Clarity of minor themes | Were minor themes or variations described? | Yes. 13 subthemes (minor themes/variations) derived from the four major themes were comprehensively described in the manuscript. All subthemes were sorted systematically, supplemented with original interview excerpts to reflect different attitudes, barriers and facilitators of older adults toward generative AI. |

**Supplementary File:2**

**Interview guide on experiences of generative ai use in navigating complex health decisions among older adults with multimorbidity: a qualitative study**

**Opening**

Thank you for participating in this interview. We are conducting a study on how older adults use intelligent conversational tools (such as Kimi, doubao, deepseek.) to assist with health decision-making. Your experience is extremely valuable. The interview content will only be used for academic research and kept strictly confidential. You may choose not to answer any questions at any time. The whole interview will last about 45–60 minutes.

**1. Please describe your main current health problems and how these issues affect your daily life.**

- Follow-up: When you face multiple health problems at the same time, have you ever received advice from doctors that confused you or made it difficult to choose? Can you give a specific example?

**2. When did you first use AI to consult health-related questions? What situation prompted you to do so at that time?**

- Follow-up 1: What did you hope to obtain from AI? (Disease explanation, medication advice, medical guidance, psychological reassurance)
- Follow-up 2: Compared with asking doctors, family members, or searching online, what unique advantages or disadvantages do you think asking AI has?

3. In what specific health decision-making contexts do you choose to ask AI? (Such as adjusting medications, choosing examinations, referral decisions, lifestyle changes)

- Follow-up 1: For which health problems do you think AI “makes reasonable points”? Which problems do you think it is “not very reliable”?
- Follow-up 2: When AI recommendations are inconsistent with doctors’ advice, how do you handle this?

4. Describe one experience of talking with AI about health issues that impressed you most. How did you ask the question? How did AI respond?

- Follow-up 1: During the conversation, did you need to adjust your way of asking to make AI understand? (Such as simplifying language, asking step by step, supplementing medical history)
- Follow-up 2: Do you think AI understands the special needs of you as an older adult? (Multimorbidity, memory decline, operational inconvenience)

5. Have you encountered any difficulties or frustrations when using AI to search for health information?

- Follow-up 1: Are these difficulties at the technical operation level (typing, interface) or at the content comprehension level (terminology, length of answers)?
- Follow-up 2: Have these difficulties affected your willingness to continue using AI? How did you overcome them?

6. Does anyone help you use AI (such as children, spouse)? How does this help affect your experience?

- Follow-up 1: Without help from others, do you think you could use AI independently? Why?
- Follow-up 2: What attitude do your family members hold toward your use of AI? (Supportive, worried, indifferent) How does this attitude influence your usage?

7. How do you evaluate your ability to use AI? (Such as finding information, judging answer quality, using it when communicating with doctors)

- Follow-up: Do you worry about over-relying on AI? Where does this concern come from?

8. After talking with AI, have you felt any changes in your role in health decision-making?

- Follow-up 1: Has AI made you more “confident” when communicating with doctors? Can you give an example?
- Follow-up 2: Have you ever shown AI answers to doctors? How did the doctors react? How did this affect the doctor-patient relationship?

9. When facing complex decisions (such as surgery, changing treatment plans), what is the specific position of AI in your decision-making process?

- Follow-up 1: How do you integrate information from AI, doctors, family members, and other sources?
- Follow-up 2: When AI recommendations are vague or offer multiple options, do you feel more anxious or more in control?

10. Looking back on your overall experience, what has using AI brought you? (Positive / Negative)

- Follow-up: If you used one sentence to describe your “relationship” with AI, what would you say? (Tool, partner, advisor, stranger)

11. What improvements do you think AI needs to better help older adults with complex health decisions?

- Follow-up: If medical resources were more sufficient and seeing specialists were easier, would you still choose to ask AI? Why?

**Closing statement:**

Thank you very much for sharing these valuable experiences. Your statements are very important for us to understand how older adults interact with AI. We will organize the interview records later. If you are willing to check the content or supplement ideas, we can contact you by phone or email. Thank you again for your participation!

**Supplementary File:3**

**A summary of the thematic structure**

| Themes | Subthemes |
| --- | --- |
| 1. Perceived Usefulness: AI as a Digital Advisor for Health Decision-Making | 1.1 *Initial Situational Triggers for First Generative AI Health Inquiry* |
|  | 1.2 *Instantaneity and Convenience of Information Access* |
|  | 1.3 *Integration of Multisource Information and Personalized Explanation* |
|  | 1.4 *Enhanced Decision-Making Confidence and Empowerment in Medical Communication* |
| 2. Perceived Ease of Use: Threshold Effects in Crossing the Digital Divide | 2.1 *Age‑Related Challenges in Interface Operation and Interaction Design* |
|  | 2.2 *Complexity of Information Input and Query Strategies* |
|  | 2.3 *Dynamic Game Between Technology Anxiety and Self‑Efficacy* |
| 3. Resource Mobilization: Synergy Between Personal and Social Support Systems | 3.1 *ehealth Literacy and Critical Evaluation Ability* |
|  | 3.2 *Family Digital Support and Tensions in Intergenerational Support* |
|  | 3.3 *Healthcare Professional Guidance and Accessibility of Community Support* |
| 4. Behavioral Intention and Actual Use: Evolutionary Trajectory from Trial Adoption to Sustained Dependence | 4.1 *Formation and Reinforcement of Positive Attitudes Toward Use* |
|  | 4.2 *Inhibiting Path Triggered by Technology Anxiety and Algorithm Aversion* |
|  | 4.3 *Differentiation and Context Dependence of Usage Patterns* |
